# *Caenorhabditis* Intervention Testing Program: the putative mTOR inhibitors Cinnarizine and Meclizine do not extend lifespan in *C. elegans*

**DOI:** 10.17912/micropub.biology.002198

**Published:** 2026-09-14

**Authors:** Erik Johnson, Christine A. Sedore, Anna L. Coleman-Hulbert, Brian D. Onken, Vijaya Madhuri Achanta, Yuhua Song, Gino Cortopassi, Gordon J. Lithgow, Monica Driscoll, Patrick C. Phillips

**Affiliations:** 1 Institute of Ecology and Evolution, University of Oregon, Eugene, Oregon, USA; 2 Department of Molecular Biology and Biochemistry, Rutgers University, Piscataway, New Jersey, USA; 3 Department of Molecular Biosciences, University of California Davis, Davis, California, USA; 4 The Buck Institute for Research on Aging, Novato, California, USA

## Abstract

The mechanistic target of rapamycin (mTOR), a protein kinase and master cell regulator, is one of the most validated longevity drug targets: mTOR inhibition by compounds like rapamycin has been shown to significantly extend lifespan in numerous model organisms. Here, we tested whether the novel putative mTOR-inhibiting compounds cinnarizine and meclizine could likewise increase lifespan in the nematode *C. elegans* , following standardized protocols from the *Caenorhabditis* Intervention Testing Program (CITP). Our results indicate that cinnarizine and meclizine have no effect on *C. elegans* lifespan at lower doses, and that both compounds exert a toxic effect at higher doses, significantly shortening lifespan.

**Figure 1. Longevity of C. elegans under adult drug exposure:** (A) Survival curves for *C. elegans* strain N2 exposed to 0, 1, 3, 10, 30, and 100 µM cinnarizine (orange) or meclizine (green) starting on the first day of adulthood, measured in a single lab (University of Oregon). (B) The highest non-toxic dose for both compounds (3 µM) replicated across two labs (Oregon and Rutgers). Each dot represents the percent change in median lifespan of a single compound plate as compared to its specific control. The shape indicates the lab in which the replicate was tested (circles: Oregon; diamonds: Rutgers). The bars represent the mean and standard error of the mean. All statistical comparisons were made with a Cox proportional hazards (CPH) mixed-model using the coxme v.2.2-22 package in R. Asterisks represent *p* -values from the CPH model such that **** *p* <.0001, *** *p* <.001, ** *p* <.01, and * *p* <.05.

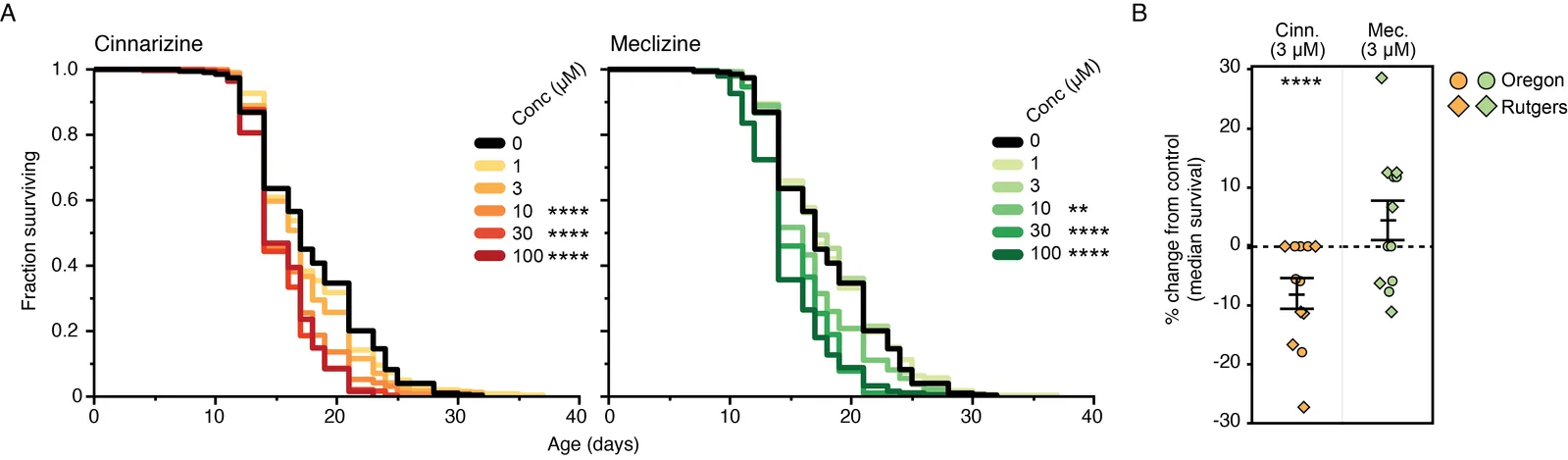



## Description

The *Caenorhabditis* Intervention Testing Program (CITP) is a multi-institute research consortium with the aim of identifying compounds that robustly extend lifespan with reproducible effects across genetically diverse *Caenorhabditis* species and strains (Lucanic et al., 2017). Prioritization of compounds for testing is based on recommendations made by our scientific Access Panel and the CITP Steering Committee; nominations for compounds to test can be made by any individual, nonprofit, academic group, or business during the CITP's annual call-for-submissions period (https://citpaging.org/submissions). Several factors, such as predicted or known interactions with established lifespan-regulating pathways, computational predictions for effects on lifespan or health-span (Coleman-Hulbert et al., 2019), or previous reports of life- or health-span extension in laboratory animals are considered when evaluating a compound for testing in the CITP pipeline.

Both cinnarizine and meclizine are piperazine-based antihistamines. Clinically, both are used in the treatment of nausea and vomiting associated with motion sickness and in the treatment of vestibular disorders such as vertigo (Pianese et al., 2002; Patel et al., 2011). Like the well-characterized lifespan-extending compound rapamycin, cinnarizine and meclizine were also both recently identified in a screen of more than 1600 human medicines targeting mTORC1-specific inhibitors (Allen et al., 2018). The mTOR kinase forms two complexes, mTORC1 and mTORC2, which are composed of discrete protein binding partners that are sensitive to distinct stimuli (Panwar et al., 2023). Numerous studies have found that genetic inhibition of mTORC1 or its downstream signaling pathways extends lifespan (Vellai et al., 2003; Kaeberlein et al., 2005; Lamming et al., 2013), whereas inhibition of mTORC2 has been associated with decreased lifespan in worms and mice (Lamming et al., 2012; Lamming et al., 2014). Both cinnarizine and meclizine were recently shown to bind to mTOR and significantly inhibit mTORC1, measured as phospho-S6 kinase inhibition, but not mTORC2, determined by the inability to inhibit phospho-Akt (Allen et al., 2018; Sandoval et al., 2020), raising the possibility that, like rapamycin, these novel mTORC1 inhibitors might also extend lifespan in model organisms, yet with fewer side effects than rapamycin. Of note, identification and classification of these compounds as mTOR inhibitors is inferred using human and mouse mTOR, and the specific inhibition of *C. elegans* mTOR/ LET-363 is unknown, thus both compounds should be considered putative mTOR inhibitors in *C. elegans* . Regardless, mTOR is highly conserved in nematodes (Blackwell et al., 2019), and cinnarizine was shown to increase longevity in *C. elegans* (Ye et al., 2014). Additionally, the mouse Intervention Testing Program (ITP) found that meclizine extends lifespan in male mice (Harrison et al., 2023). Cinnarizine and meclizine have a smaller size (369 g/mol and 391 g/mol, respectively) compared to rapamycin (914 g/mol). They are approved for use in humans (cinnarizine since 1955 and meclizine since 1953) and display a relatively mild profile of known side-effects ( *e.g.* drowsiness and dry mouth) as compared to the more serious adverse immunosuppressive effects in patients treated with rapamycin (Kraig et al., 2017). Although cinnarizine has also been associated with drug-induced parkinsonism (Martí-Massó & Poza, 1998; Terland & Flatmark, 1999), we were interested in testing the longevity effects of both compounds in *C. elegans* .

Here, we tested whether cinnarizine and meclizine could reproducibly and robustly increase lifespan in *C. elegans* using our recently revised workflow. *C. elegans* N2 worms were assayed on five different concentrations of cinnarizine and meclizine at a single research site (University of Oregon). Both cinnarizine and meclizine had no effect on lifespan at the two lowest doses tested (1 µM and 3 µM, along with 10 µM for meclizine alone; Fig. 1A ). Conversely, both compounds exerted a toxic effect at the higher concentrations tested (10, 30, and 100 µM for cinnarizine and 30 and 100 µM for meclizine), significantly shortening lifespan ( Fig. 1A ). Following the CITP protocol for null or negative results, the experiment was repeated at the highest non-toxic dose (3 µM) for both compounds at a second research site (Rutgers University); results from this assay demonstrated that 3 µM of meclizine at both the Rutgers and Oregon sites had no significant effect, while treatment with 3 µM of cinnarizine showed a significantly negative lifespan effect when the data from both sites were pooled ( Fig. 1B ).

Overall, our preliminary findings may seem surprising given the observed pro-longevity effects of the mTORC-1 inhibiting compound rapamycin in numerous model organisms (Mannick & Lamming, 2023). However, previous CITP studies on rapamycin also showed a null effect on lifespan in *C. elegans* (Banse et al., 2024) underscoring the consistency of the current results. This fact, combined with results from other studies showing the ability of rapamycin to extend life in *C. elegans* , indicates that cinnarizine and meclizine's potential ability to extend lifespan may be dependent on the specific protocols used, and further experimentation is likely warranted. The absence of lifespan extension here may indicate that mTOR inhibition does not confer pro-longevity effects in this context. Alternatively, the lack of an observed effect could reflect limited compound uptake and/or exposure in the target tissues, metabolism or efflux, or inadequate activity against mTOR in *C. elegans* . Others in the field acknowledge the effect of assay conditions on outcomes of aging interventions, including those who previously reported a positive effect from cinnarizine on *C. elegans* lifespan (Ye et al., 2014). Given the aims of the CITP to identify compounds that robustly and reproducibly increase lifespan (with an effect size of 20% or more) following a standardized pipeline, and the fact that cinnarizine and meclizine showed null or negative lifespan effects using our protocol, further investigation under the CITP workflow was not pursued.

## Methods

For all experiments, *C. elegans* N2 worms were age-synchronized by timed egg-lays on standard 60 mm diameter Nematode Growth Media (NGM) plates and transferred at a density of 50 individuals per 35 mm treated plate in triplicate upon onset of adulthood (for control plates, there were six replicates of 50 animals each). Both cinnarizine and meclizine were dissolved in DMSO and diluted appropriately such that addition of 132.5 µl of solution to 35 mm diameter plates containing NGM with lawns of *E. coli* OP50-1 and 51 µm FUdR would generate the following final concentrations: 0 µM (control), 1 µM, 3 µM, 10 µM, 30 µM, and 100 µM. Final concentration of DMSO in all plates was 0.25%. Animals were maintained at 20 °C and 80% RH and moved to fresh plates on the first, second, and fifth day of adulthood, then once weekly afterward. Three times weekly, we observed animals for spontaneous movement or movement after gentle perturbation with a 0.2 mm diameter platinum wire. Death was scored as a lack of movement.

Statistical analyses were performed as previously described (Lucanic et al. 2017). In brief, survival was analyzed both with a generalized linear model using the lme4 package (version 1.1-35.5), and with a mixed-model Cox proportional hazards (CPH) using the coxme package (version 2.2-22; Therneau 2020) in the R statistical language (R Core Team 2021; version 4.4.1). Compound effects were analyzed as a planned comparison between individuals exposed to compound (cinnarizine or meclizine) or vehicle control (DMSO) (multcomp package, version 1.4-26). The raw data can be accessed on the CITP Data Portal (citpaging.org/portal v2.1), and on figshare.com along with CITP SOPs and the R scripts used for analysis (https://doi.org/10.6084/m9.figshare.c.7561101).

## Reagents

Experiments were performed using *C. elegans* N2 _ PD1073 (Banse et al. 2019; Yoshimura et al. 2019) from the CGC, which is funded by NIH Office of Research Infrastructure Programs (P40 OD010440). For chemical interventions, cinnarizine (Alfa Aesar CAS: 298-57-7) and meclizine (Millipore Sigma CAS: 1104-22-9) were obtained in solid form and dissolved in DMSO (Sigma-Aldrich).

## References

[R1] Allen SA, Tomilov A, Cortopassi GA (2018). Small molecules bind human mTOR protein and inhibit mTORC1 specifically.. Biochem Pharmacol.

[R2] Banse SA, Lucanic M, Sedore CA, Coleman-Hulbert AL, Plummer WT, Chen E, Kish JL, Hall D, Onken B, Presley MP, Jones EG, Blue BW, Garrett T, Abbott M, Xue J, Guo S, Johnson E, Foulger AC, Chamoli M, Falkowski R, Melentijevic I, Harinath G, Huynh P, Patel S, Edgar D, Jarrett CM, Guo M, Kapahi P, Lithgow GJ, Driscoll M, Phillips PC (2019). Automated lifespan determination across Caenorhabditis strains and species reveals assay-specific effects of chemical interventions.. Geroscience.

[R3] Banse SA, Sedore CA, Johnson E, Coleman-Hulbert AL, Onken B, Hall D, Jackson EG, Huynh P, Foulger AC, Guo S, Garrett T, Xue J, Inman D, Morshead ML, Plummer WT, Chen E, Bhaumik D, Chen MK, Harinath G, Chamoli M, Quinn RP, Falkowski R, Edgar D, Schmidt MO, Lucanic M, Guo M, Driscoll M, Lithgow GJ, Phillips PC (2023). Antioxidants green tea extract and nordihydroguaiaretic acid confer species and strain-specific lifespan and health effects in Caenorhabditis nematodes.. Geroscience.

[R4] Blackwell TK, Sewell AK, Wu Z, Han M (2019). TOR Signaling in Caenorhabditis elegans Development, Metabolism, and Aging.. Genetics.

[R5] Harrison DE, Strong R, Reifsnyder P, Rosenthal N, Korstanje R, Fernandez E, Flurkey K, Ginsburg BC, Murrell MD, Javors MA, Lopez-Cruzan M, Nelson JF, Willcox BJ, Allsopp R, Watumull DM, Watumull DG, Cortopassi G, Kirkland JL, Tchkonia T, Choi YG, Yousefzadeh MJ, Robbins PD, Mitchell JR, Acar M, Sarnoski EA, Bene MR, Salmon A, Kumar N, Miller RA (2023). Astaxanthin and meclizine extend lifespan in UM-HET3 male mice; fisetin, SG1002 (hydrogen sulfide donor), dimethyl fumarate, mycophenolic acid, and 4-phenylbutyrate do not significantly affect lifespan in either sex at the doses and schedules used.. Geroscience.

[R6] Kaeberlein M, Powers RW 3rd, Steffen KK, Westman EA, Hu D, Dang N, Kerr EO, Kirkland KT, Fields S, Kennedy BK (2005). Regulation of yeast replicative life span by TOR and Sch9 in response to nutrients.. Science.

[R7] Kraig E, Linehan LA, Liang H, Romo TQ, Liu Q, Wu Y, Benavides AD, Curiel TJ, Javors MA, Musi N, Chiodo L, Koek W, Gelfond JAL, Kellogg DL Jr (2018). A randomized control trial to establish the feasibility and safety of rapamycin treatment in an older human cohort: Immunological, physical performance, and cognitive effects.. Exp Gerontol.

[R8] Lamming DW, Ye L, Katajisto P, Goncalves MD, Saitoh M, Stevens DM, Davis JG, Salmon AB, Richardson A, Ahima RS, Guertin DA, Sabatini DM, Baur JA (2012). Rapamycin-induced insulin resistance is mediated by mTORC2 loss and uncoupled from longevity.. Science.

[R9] Lamming DW, Ye L, Sabatini DM, Baur JA (2013). Rapalogs and mTOR inhibitors as anti-aging therapeutics.. J Clin Invest.

[R10] Lamming DW, Mihaylova MM, Katajisto P, Baar EL, Yilmaz OH, Hutchins A, Gultekin Y, Gaither R, Sabatini DM (2014). Depletion of Rictor, an essential protein component of mTORC2, decreases male lifespan.. Aging Cell.

[R11] Lucanic M, Plummer WT, Chen E, Harke J, Foulger AC, Onken B, Coleman-Hulbert AL, Dumas KJ, Guo S, Johnson E, Bhaumik D, Xue J, Crist AB, Presley MP, Harinath G, Sedore CA, Chamoli M, Kamat S, Chen MK, Angeli S, Chang C, Willis JH, Edgar D, Royal MA, Chao EA, Patel S, Garrett T, Ibanez-Ventoso C, Hope J, Kish JL, Guo M, Lithgow GJ, Driscoll M, Phillips PC (2017). Impact of genetic background and experimental reproducibility on identifying chemical compounds with robust longevity effects.. Nat Commun.

[R12] Mannick JB, Lamming DW (2023). Targeting the biology of aging with mTOR inhibitors.. Nat Aging.

[R13] Martí-Massó JF, Poza JJ (1998). Cinnarizine-induced parkinsonism: ten years later.. Mov Disord.

[R14] Panwar V, Singh A, Bhatt M, Tonk RK, Azizov S, Raza AS, Sengupta S, Kumar D, Garg M (2023). Multifaceted role of mTOR (mammalian target of rapamycin) signaling pathway in human health and disease.. Signal Transduct Target Ther.

[R15] Patel Priti N., Ambizas Emily M. (2011). Meclizine: Safety and Efficacy in the Treatment and Prevention of Motion Sickness. Clinical Medicine Insights: Therapeutics.

[R16] Pianese CP, Hidalgo LO, González RH, Madrid CE, Ponce JE, Ramírez AM, Morán LM, Arenas JE, Rubio AT, Uribe JO, Abiuso J, Hanuch E, Alegría J, Volpi C, Flaskamp R, Sanjuán AP, Gómez JM, Hernández J, Pedraza A, Quijano D, Martínez C, Castañeda JR, Guerra OJ, F GV (2002). New approaches to the management of peripheral vertigo: efficacy and safety of two calcium antagonists in a 12-week, multinational, double-blind study.. Otol Neurotol.

[R17] R Core Team, 2021 R: A language and environment for statistical computing. R Foundation for Statistical Computing, Vienna, Austria. URL https://www.R-project.org/.

[R18] Sandoval JA, Tomilov A, Datta S, Allen S, O'Donnell R, Sears T, Woolard K, Kovalskyy D, Angelastro JM, Cortopassi G (2020). Novel mTORC1 Inhibitors Kill Glioblastoma Stem Cells.. Pharmaceuticals (Basel).

[R19] Terland O, Flatmark T (1999). Drug-induced parkinsonism: cinnarizine and flunarizine are potent uncouplers of the vacuolar H+-ATPase in catecholamine storage vesicles.. Neuropharmacology.

[R20] Therneau T. M., 2020 coxme: Mixed Effects Cox Models. R package version 2.2.16.

[R21] Vellai T, Takacs-Vellai K, Zhang Y, Kovacs AL, Orosz L, Müller F (2003). Genetics: influence of TOR kinase on lifespan in C. elegans.. Nature.

[R22] Ye X, Linton JM, Schork NJ, Buck LB, Petrascheck M (2013). A pharmacological network for lifespan extension in Caenorhabditis elegans.. Aging Cell.

